# Supplementary material for: Identification of photoperiod-induced specific miRNAs in the adrenal glands of Sunite sheep (Ovis aries)
Source: Front Vet Sci. 2022 Jul 22;9:888207. doi: 10.3389/fvets.2022.888207 (PMC9354845; doi:10.3389/fvets.2022.888207)
Supplement: Supplementary file 1 [file Data_Sheet_1.ZIP › Supplementary file/miRDeep2 file.zip!/miRDeep2 file/Know_Structure/LP42A2/oar-mir-3958.pdf]

miRBase precursor : oar-mir-3958
Total read count : 15346
oar-miR-3958-5p read count : 13
oar-miR-3958-3p read count : 15328
remaining reads : 5

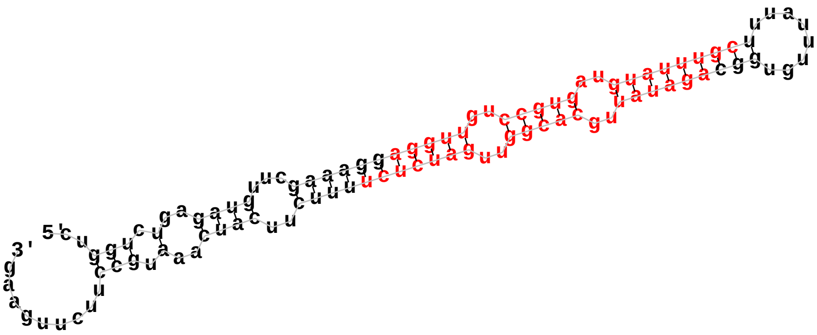

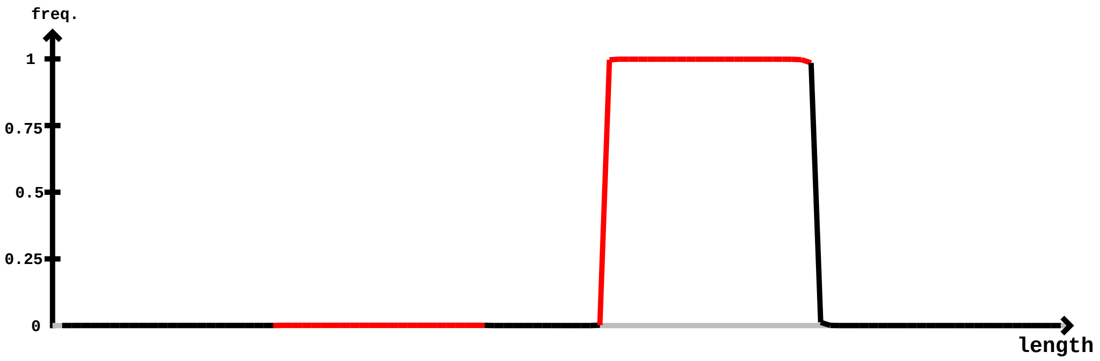

oar-miR-3958-3p
oar-miR-3958-5p

```
5'- cuggucugagauguucgaaaggagguuguccgugauguauuugcuuuauuuguggcagauauugcacgguugaucucuuuucuucaucaaaugccuucuugaag   -3'   exp
    ..(((.(..((((...((((((((((..(((((..((((((((........))))))))..)))))..))))))))))..))))..).)))............   reads   mm   sample
    cuggucugagauguucgaaagg.................................................................................   3       0    seq
    .uggucugagauguucgaaagg.................................................................................   1       0    seq
    ......................agguuguccgugauguauuugc...........................................................   12      0    seq
    ......................agguuguccgugauguauuugcuuu........................................................   1       0    seq
    ............................uccgugauguauuugcuuuauuu....................................................   1       0    seq
    .......................................................cagauauugcacgguugaucu...........................   2       0    seq
    .......................................................cagauauugcacgguugaucuc..........................   2       0    seq
    .......................................................cagauauugcacgguugaucucu.........................   19      0    seq
    ........................................................agauauugcacgguugau.............................   2       0    seq
    ........................................................agauauugcacgguugauc............................   3       0    seq
    ........................................................agauauugcacgguugaucu...........................   23      0    seq
    ........................................................agauauugcacgguugaucuc..........................   179     0    seq
    ........................................................agauauugcacgguugaucucu.........................   14876   0    seq
    ........................................................agauauugcacgguugaucucuu........................   177     0    seq
    ........................................................agauauugcacgguugaucucuuu.......................   9       0    seq
    .........................................................gauauugcacgguugaucucu.........................   36      0    seq
```
